# Efficacy and safety of different immunosuppressive agents in patients with calcineurin inhibitor-dependent primary membranous nephropathy

**DOI:** 10.3389/fmed.2026.1865720

**Published:** 2026-06-17

**Authors:** Zongying Li, Shuyi Cao, Zheng Liang, Fei Zhao, Kang Li

**Affiliations:** Department of Nephrology, Cangzhou Central Hospital, Cangzhou, Hebei, China

**Keywords:** calcineurin inhibitor dependence, conversion therapy, cyclophosphamide, primary membranous nephropathy, rituximab, tripterygium glycosides

## Abstract

**Objective:**

To compare the efficacy and safety of rituximab (RTX), cyclophosphamide (CTX), and tripterygium glycosides (TII) as conversion therapies in patients with calcineurin inhibitor (CNI)–dependent primary membranous nephropathy (PMN), and to evaluate their ability to induce clinical and immunological remission and facilitate CNI withdrawal.

**Methods:**

Seventy-nine patients with CNI-dependent primary membranous nephropathy (PMN) admitted between January 2018 and June 2023 were retrospectively enrolled in this study, and divided into three groups: the RTX group (*n* = 37), the CTX group (*n* = 23), and the TII group (*n* = 19). The primary endpoints were the *de novo* complete remission rate and the complete CNI withdrawal rate at 12 months.

**Results:**

After 12 months of treatment, among patients who did not achieve complete remission at baseline, the rates of newly achieved complete remission and CNI withdrawal were significantly higher in the RTX and CTX groups than in the TII group (all *p* < 0.05). The immunological remission rate was significantly higher in the RTX group than in the TII group (*p* < 0.05). Urinary protein was significantly reduced and serum albumin was increased in all three groups, with stable renal function. The overall adverse event rate in the RTX group (8.11%) was significantly lower than that in the CTX group (30.43%) and the TII group (31.58%, all *p* < 0.05).

**Conclusion:**

RTX and CTX are equally effective in treating CNI-dependent PMN, both significantly improving clinical complete remission rates and enabling successful CNI discontinuation. RTX shows advantages in achieving deeper immunological remission and better safety. TII can help reduce proteinuria and facilitate CNI tapering, making it suitable for patients intolerant to intensive immunosuppression or seeking a lower-risk treatment option.

## 1 Introduction

Membranous nephropathy (MN) is one of the most common pathological types of nephrotic syndrome in adults. Calcineurin inhibitors (CNIs), including cyclosporine and tacrolimus, are recommended as first-line immunosuppressive therapies (1). They achieve high remission rates during induction, yet the post-withdrawal recurrence rate reaches 38.5–50%, which is substantially higher than that of cyclophosphamide (4.3%) and rituximab (3.4%) (2, 3). High relapse rates often result in long-term maintenance therapy (4–7), which increases the risk of nephrotoxicity, hypertension, diabetes mellitus and other adverse reactions, while placing heavy economic burdens on patients (1, 2, 8–10). Therefore, safely tapering CNIs while maintaining remission has significant clinical implications. In recent years, rituximab (RTX) has provided a new strategy for PMN treatment by depleting pathogenic B cells and suppressing autoantibody production, thereby achieving more durable remission (11, 12).

Cyclophosphamide (CTX) combined with glucocorticoids (GC) is a classic treatment regimen with high remission rates (13), and remains the preferred therapy for high-risk and very high-risk PMN patients (1), although it is associated with numerous adverse effects (14, 15). Tripterygium glycosides (TII) is a commonly used traditional Chinese medicine with immunosuppressive effects, and has been proven effective in the treatment of membranous nephropathy (16–18). Currently, it remains unclear the optimal treatment for CNI-dependent PMN patients to safely taper and discontinue CNIs while sustaining complete clinical and immunological remission.

Accordingly, this study retrospectively compared the efficacy and safety of three conversion regimens—RTX, CTX, and TII—in patients with CNI-dependent PMN, aiming to provide evidence for optimizing clinical treatment strategies.

## 2 Subjects and methods

### 2.1 Study subjects

Patients with PMN diagnosed and treated in the Nephrology Department of our hospital from January 2018 to June 2023 were retrospectively enrolled.

#### 2.1.1 Inclusion criteria

- Diagnosed with MN by renal biopsy;
- CNI dependence as defined in this study: after achieving clinical remission with CNI therapy, proteinuria recurs within 6 months following a dose reduction (≥25%) or discontinuation of the drug (defined as a 24-h urinary protein level exceeding 50% above the remission level and an absolute value >3.5 g), but proteinuria is recontrolled upon resuming the original CNI dose.

#### 2.1.2 Exclusion criteria

- Patients with secondary membranous nephropathy complicated with autoimmune diseases (such as systemic lupus erythematosus, Sjögren’s syndrome), viral hepatitis (hepatitis B, hepatitis C), malignant tumors and other conditions;
- Patients with concurrent active infection or severe dysfunction of the heart, liver or kidney.
- Pregnant or lactating women;
- Incomplete follow-up data.

### 2.2 Research methods and data collection

Patients were divided into 3 groups according to treatment regimens: RTX group: received RTX combined with CNI plus low-dose glucocorticoids; CTX group: intravenous CTX combined with CNI plus low-dose glucocorticoids; TII group: oral tripterygium glycosides combined with CNI plus low-dose glucocorticoids.

#### 2.2.1 Baseline clinical characteristics

Age, sex, duration of CNI treatment, and baseline disease remission status, as shown in Table 1.

**Table 1:** Comparison of baseline characteristics among three groups of patients.

Variable	Total (*n* = 79)	RTX group (*n* = 37)	CTX group (*n* = 23)	TII group (*n* = 19)	*p*-value
Male, *n* (%)	49 (62.03%)	19 (51.35%)	15 (65.22%)	15 (78.95%)	0.123
Age, years	40 (39–52)	39 (35–40)	52 (51–55)	52 (51–53)	<0.001^△,#^
24hUTP, g	1.2 (0.19–2.14)	1.18 (0.87–2.01)	1.36 (0.13–2.93)	1.05 (0.18–2.94)	0.137
Albumin, g/L	36.08 ± 5.22	34.96 ± 5.19	35.65 ± 5.36	38.93 ± 5.22	0.385
Globulin, g/L	23.31 ± 2.80	23.19 ± 2.72	23.72 ± 3.32	23.04 ± 2.25	0.892
Serum creatinine, μmol/L	60.15 ± 10.26	57.86 ± 11.15	65.10 ± 11.71	62.79 ± 8.70	0.487
eGFR, mL·min^−1^ (1.73 m^2^)^−1^	108.44 ± 11.75	113.44 ± 12.34	103.59 ± 8.19	102.92 (98.59–108.35)	0.393
Anti-PLA2R positive, *n* (%)	40 (50.63%)	20 (54.05%)	12 (52.17%)	8 (42.11%)	0.688
Anti-PLA2R ≤ 2 U/mL, *n* (%)	25 (31.65%)	13 (35.14%)	7 (30.43%)	5 (26.32%)	0.789
B-cell count, /μL	–	299 (191.5–462)	–	–	–
CNI treatment duration, months	22.33 ± 6.07	22.16 ± 4.54	19 (15–20)	26.26 ± 4.85	<0.001^#,※^
Baseline CR rate, *n* (%)	30 (37.97%)	14 (37.83%)	9 (39.13%)	7 (36.84%)	0.988

^△^*p* < 0.05 RTX vs CTX; ^#^*p* < 0.05 RTX vs TII; ^※^*p* < 0.05 CTX vs TII.

#### 2.2.2 Baseline laboratory data

24-h urinary protein (24hUTP), serum albumin (ALB), globulin (GLB), serum creatinine (Scr), estimated glomerular filtration rate (eGFR), anti-phospholipase A2 receptor antibody (anti-PLA2R antibody), and B-cell count (RTX group), as shown in Table 1.

#### 2.2.3 Collection of follow-up data

24-h urinary total protein (24hUTP), albumin (ALB), serum creatinine (Scr) and estimated glomerular filtration rate (eGFR); clinical and immunological remission status; calcineurin inhibitor (CNI) dose reduction and withdrawal status; and incidence of adverse events.

### 2.3 Study endpoints and definitions

Primary endpoints: At 12 months of treatment: ① Rate of new complete response; ② Rate of complete calcineurin inhibitor (CNI) withdrawal (defined as permanent discontinuation without disease recurrence).

Secondary endpoints: ① the rates of new complete remission (CR) and CNI withdrawal at 6 months; ② the rate of new immunological remission at 6 and 12 months; ③ changes in 24hUTP, ALB, Scr, and eGFR levels before and after treatment; ④ the incidence of adverse events (AE).

Indicator Definition: (1) Remission criteria were established according to the KDIGO guidelines (1). Complete Remission (CR) was defined as 24-h urinary total protein (24hUTP) < 0.3 g accompanied by normal serum albumin levels. Partial Remission (PR) was defined as a ≥ 50% reduction in urinary protein from baseline and an absolute 24-h urinary protein level < 3.5 g. Total Remission (TR) included both CR and PR. (2) Anti-PLA2R antibody positivity: serum titer ≥ 14 RU/mL; Complete Immunological remission: serum anti-PLA2R antibody titer ≤ 2 RU/mL (repeated testing after 2–4 weeks for confirmation) (1). (3) eGFR was calculated using the CKD-EPI 2021 equation (without race coefficient). (4) Complete relapse: 24-h urinary protein level >3.5 g after remission; Partial relapse: increased proteinuria after remission, with 24-h urinary protein quantification between 0.3 and 3.5 g/d. (5) Overcoming Drug Dependence: Complete cessation of calcineurin inhibitor (CNI) therapy during clinical remission. (6) Endpoint event: renal dysfunction (eGFR decrease > 30% from baseline or eGFR <15 mL·min^−1^·(1.73 m^2^)^−1^), end-stage renal disease (ESRD), or death during follow-up. (7) Serious adverse event: clinical death, severe pulmonary infection, pulmonary embolism, cerebral infarction, myocardial infarction, or hospitalization due to adverse events.

### 2.4 Statistical analysis

All statistical analyses were conducted with SPSS 31.0. Normally distributed continuous data were presented as mean ± SD and analyzed by one-way ANOVA. Non-normally distributed data were described as median (IQR) and compared using the Kruskal–Wallis *H* test. Categorical data were shown as *n* (%) and assessed with the chi-square test or Fisher’s exact test. The Bonferroni correction was applied for pairwise comparisons. A *p* value < 0.05 indicated statistical significance.

## 3 Results

### 3.1 Baseline characteristics

A total of 79 CNI-dependent primary membranous nephropathy (PMN) patients were enrolled in this study. For the enrolled patients, the CNI medication regimens were as follows: 31 cases received tacrolimus, and 48 cases received cyclosporine. The initial dosage for all patients followed the KDIGO guidelines: tacrolimus was administered at 0.05–0.1 mg/kg/d with the trough concentration of 3–8 ng/mL, and cyclosporine was administered at 3.5 mg/kg/d with the trough concentration of 125–225 ng/mL.

According to the treatment regimen, the patients were divided into the RTX group (*n* = 37), the CTX group (*n* = 23) and the TII group (*n* = 19). No statistically significant differences were observed among the three groups in gender, baseline 24hUTP, ALB, GLB, Scr, eGFR, anti-PLA2R positive rate, baseline CR rate, or baseline immunological remission rate among the three groups (all *p* > 0.05). Age was significantly lower in the RTX group [39 (35–40) years] than in the CTX [52 (51–55) years] and TII [52 (51–53) years] groups (both *p* < 0.05). The duration of CNI treatment was significantly shorter in the RTX [22.16 ± 4.54 months] and CTX [19 (15–20) months] groups than in the TII group [26.26 ± 4.85 months] (both *p* < 0.05) (Table 1).

### 3.2 Treatment regimens

RTX group (*n* = 37). Patients received RTX combined with CNI and low-dose glucocorticoids. Dosing regimens were stratified by early remission status: ① CR patients (*n* = 14): reduced-dose RTX. 5 cases received 0.5 g × 2 doses, 8 cases received 0.5 g × 3 doses, and 1 case received 1 g × 1 dose. All sustained remission at 3 and 6 months. ② PR patients (*n* = 23): standard-dose RTX. 16 cases received 0.5 g × 4 doses, and 7 cases received 1 g every 2 weeks for 2 doses.

At 6 months, retreatment was determined based on anti-PLA2R antibody levels and clinical status. A total of 28 patients received retreatment: 17 with 0.5 g × 1 dose and 11 with 1 g × 1 dose. Regular reassessments were performed every 6 months (Table 2).

**Table 2:** Dosing regimens for patients receiving rituximab therapy.

Regimen	Initial treatment (*n* = 37)	Treatment at 6 months (*n* = 28)
RTX 375 mg/m^2^ weekly × 1	0	17
RTX 375 mg/m^2^ weekly × 2	5	0
RTX 375 mg/m^2^ weekly × 3	8	0
RTX 375 mg/m^2^ weekly × 4	16	0
RTX 1 g every 2 weeks × 1	1	11
RTX 1 g every 2 weeks × 2	7	0

CTX group (*n* = 23). Patients received intravenous CTX at 0.6–0.8 g/month for 6 months, combined with CNI and low-dose glucocorticoids. Two patients had extended treatment intervals due to pulmonary infection.

TII group (*n* = 19). Patients received oral tripterygium glycosides 20 mg three times daily, combined with CNI and low-dose glucocorticoids. All patients underwent gradual CNI tapering.

### 3.3 Clinical remission and CNI withdrawal

Among patients without baseline CR, the new CR rates at 12 months were 73.91, 78.57, and 25.00% in the RTX, CTX, and TII groups, respectively. The rates in the RTX and CTX groups were significantly higher than that in the TII group (both *p* < 0.05). Among anti-PLA2R-positive patients, the immunological remission rates at 12 months were 79.17% (19/24), 56.25% (9/16), and 14.29% (2/14) in the RTX, CTX, and TII groups, respectively. The immunological remission rate was significantly higher in the RTX group than in the TII group (79.17% vs. 14.29%, *p* < 0.05), whereas no significant difference was found between the RTX and CTX groups (79.17% vs. 56.25%, *p* > 0.05). The rate in the RTX group was significantly higher than that in the TII group (*p* < 0.05), with no significant difference between the RTX and CTX groups (*p* > 0.05). The CNI complete withdrawal rates were 94.6, 91.3, and 15.8% in the RTX, CTX, and TII groups, respectively; rates in the RTX and CTX groups were significantly higher than that in the TII group (both *p* < 0.05). All patients without complete CNI withdrawal received dose reduction: a reduction of ≥ 50% was observed in all cases in the RTX and CTX groups, and in 9 of 16 such patients in the TII group (Table 3).

**Table 3:** Comparison of clinical remission status at 6 and 12 months of treatment.

Variable	Time	RTX	CTX	TII	*p*-value
New CR rate, *n* (%)	6 months	11 (47.83%)	3 (21.43%)	1 (8.33%)	0.041
	12 months	17 (73.91%)	11 (78.57%)	3 (25.0%)	0.010^#,※^
PR rate, *n* (%)	6 months	12 (32.43%)	11 (47.83%)	11 (57.89%)	0.163
	12 months	6 (16.22%)	3 (13.04%)	9 (47.37%)	0.020^#,※^
Total remission rate, *n* (%)	6 months	37 (100%)	23 (100%)	19 (100%)	–
	12 months	37 (100%)	23 (100%)	19 (100%)	–
New immunological remission rate, *n* (%)	6 months	10 (41.67%)	1 (6.25%)	0 (0%)	<0.001^△^
	12 months	19 (79.17%)	9 (56.25%)	2 (14.29%)	<0.001^#^
CNI withdrawal rate, *n* (%)	6 months	19 (51.35%)	2 (8.70%)	0 (0%)	<0.001^△^
	12 months	35 (94.59%)	21 (91.30%)	3 (15.78%)	<0.001^#,※^
CNI reduction ≥50%, *n*	6 months	14	8	0	–
	12 months	2	2	9	–

^△^*p* < 0.05 RTX vs CTX; ^#^*p* < 0.05 RTX vs TII; ^※^*p* < 0.05 CTX vs TII.

### 3.4 Changes in laboratory parameters

At 12 months, 24hUTP was significantly reduced in all groups. Levels were significantly lower in the RTX [0.10 (0.08–0.29) g] and CTX [0.09 (0.06–0.18) g] groups than in the TII group [0.46 (0.12–0.99) g] (both *p* < 0.05). Serum albumin returned to normal in all groups, with no significant intergroup difference (*p* = 0.552). Scr and eGFR remained stable without obvious deterioration. eGFR was significantly higher in the RTX group than in the TII group (108.86 ± 16.42 vs. 97.22 (89.97–103.18) mL·min^−1^·(1.73 m^2^)^−1^, *p* < 0.05) (Table 4).

**Table 4:** Comparison of changes in laboratory indicators among the three groups of patients after 12 months of treatment.

Variable	RTX group (*n* = 37)	CTX group (*n* = 23)	TII group (*n* = 19)	*p*-value
24hUTP (g)				
Baseline	1.18 (0.87–2.01)	1.36 (0.13–2.93)	1.05 (0.18–2.94)	0.137
12 months	0.10 (0.08–0.29)	0.09 (0.06–0.18)	0.46 (0.12–0.99)	0.002^#,※^
Intra-group comparison			Among the 10 patients in the TII group with baseline urinary protein >1 g, 6 showed a reduction of >50%.	
Serum albumin (g/L)				
Baseline	34.96 + 5.19	35.65 ± 5.36	38.93 ± 5.22	0.385
12 months	43.2 (40.85–45)	43.26 (42–44.26)	42.04 ± 3.09	0.552
Serum creatinine (μmol/L)				
Baseline	57.86 ± 11.15	65.10 ± 11.71	62.79 ± 8.70	0.487
12 months	61.29 ± 11.63	65.10 ± 11.17	68.77 ± 18.53	0.132
eGFR (mL/min/1.73m^2^)				
Baseline	113.44 ± 12.34	103.59 ± 8.19	102.92 (98.59–108.35)	0.393
12 months	108.86 ± 16.42	100.83 ± 10.44	97.22 (89.97–103.18)	0.007^#^

^△^*p* < 0.05 RTX vs CTX; ^#^*p* < 0.05 RTX vs TII; ^※^*p* < 0.05 CTX vs TII.

### 3.5 Safety analysis

During follow-up, the RTX group showed good tolerability. Two patients had infusion reactions: one with tachycardia and chest tightness, and one with rash and pruritus. Symptoms resolved after slowing infusion and antihistamine therapy.1 patient recovered from mild respiratory infection with symptomatic treatment.

In the CTX group: 5 patients had respiratory tract infections (2 with pulmonary infection requiring prolonged dosing intervals), 1 had leukopenia and 1 had liver function impairment. All recovered after treatment. All patients received hydration and alkalization, and no hemorrhagic cystitis occurred.

In the TII group: 2 patients had respiratory infections and 3 had liver function impairment, all resolved after treatment. Of 4 female patients, one 48-year-old patient had menstrual irregularity (possibly related to perimenopause). The other three were over 50 years of age and postmenopausal.

At 12 months, the overall adverse event (AE) rate was 8.1% (3/37) in the RTX group, significantly lower than 30.43% (7/23) in the CTX group and 31.6% (6/19) in the TII group (all *p* < 0.05). No treatment-related deaths or malignancies were reported.

## 4 Discussion

This study is the first to systematically compare the clinical efficacy and safety of three conversion therapies, namely rituximab (RTX), cyclophosphamide (CTX), and tripterygium glycosides (TII), in patients with calcineurin inhibitor (CNI)-dependent primary membranous nephropathy (PMN), aiming to provide evidence-based support for this challenging clinical management issue.

Regarding complete remission and CNI withdrawal rates, the RTX and CTX groups achieved high rates of complete remission and CNI cessation at 12 months of follow-up. The two regimens showed equivalent efficacy and were both significantly superior to the TII regimen. The equivalence of the two groups in the primary efficacy endpoints provides an important reference for clinicians to select individualized treatment strategies based on patient characteristics. As a major Chinese herbal medicine for kidney diseases with distinctive clinical application in China (19), tripterygium glycosides had limited efficacy in achieving complete CNI withdrawal in this study and was insufficient as a primary regimen to overcome CNI dependence.

Nevertheless, most patients in this group achieved a substantial reduction in CNI dosage, which helps minimize drug-related adverse effects. Furthermore, some patients in the TII group still achieved clinically meaningful proteinuria reduction (e.g., 60% of patients with baseline proteinuria > 1 g/d had > 50% reduction in proteinuria), which is consistent with previous reports (16, 20). These findings indicate that TII still plays a certain role in adjuvant proteinuria reduction and facilitating CNI tapering, and may be considered as an adjuvant or bridging therapy for patients with contraindications to intensive immunosuppression or those unfit for RTX/CTX treatment.

In terms of the induction of immunological remission, RTX exhibits a significantly more pronounced therapeutic effect. Anti-PLA2R antibody is a core biomarker of disease activity in primary membranous nephropathy (PMN), and its deep clearance is correlated with long-term clinical remission and reduced recurrence risk (21). Therefore, RTX may possess unique value in inducing deep immunological remission; however, whether this effect can be further translated into improved long-term prognosis still requires validation via extended follow-up.

Regarding renal outcome measures, 24-h urinary protein excretion was significantly decreased from baseline in all three groups, accompanied by a concurrent increase in serum albumin levels. Serum creatinine and estimated glomerular filtration rate (eGFR) remained stable both within and between groups, with no significant deterioration observed. Notably, the reduction in 24-h urinary protein was significantly greater in the RTX and CTX groups than in the TII group, suggesting that these two intensive immunosuppressive regimens exert a more prominent effect in reducing proteinuria. At the end of follow-up, eGFR was significantly higher in the RTX group than in the TII group. These findings indicate that RTX may confer a better renal protective effect and help maintain superior glomerular filtration function.

In terms of safety, the overall incidence of adverse events was significantly lower in the RTX group than in the CTX and TII groups. The CTX group carried characteristic risks of myelosuppression and hepatotoxicity, while liver injury and menstrual disorders were reported in the TII group (22, 23). In contrast, rituximab demonstrated a more favorable overall safety profile. For young patients, those with fertility needs, or those with multiple comorbidities, RTX may be the preferred treatment option owing to its superior safety profile while achieving comparable efficacy (1).

Several limitations of this study should be acknowledged. First, this was a single-center retrospective study with a relatively small sample size. Second, there were baseline imbalances in age and duration of CNI treatment among groups, which may have introduced bias. Third, the follow-up period was relatively short, so long-term relapse and long-term renal outcomes could not be evaluated. Further prospective, large-sample, randomized controlled trials with extended follow-up are warranted to validate the long-term benefits and risks of different therapeutic strategies.

## 5 Conclusion

In summary, for patients with CNI-dependent PMN, conversion to either RTX or CTX effectively improves the clinical complete remission rate and enables successful CNI withdrawal, with comparable efficacy between the two regimens. However, RTX offers superior advantages in inducing deep immunological remission and safety. The TII regimen has limited efficacy in facilitating complete CNI withdrawal, but can assist in reducing proteinuria and achieving CNI tapering, making it suitable for patients intolerant to intensive immunosuppression or those seeking lower-risk treatment.

## Data Availability

The original contributions presented in the study are included in the article/supplementary material, further inquiries can be directed to the corresponding author.
